# Hemodynamic derangement associated with tension pneumomediastinum during minimally invasive esophagectomy: A case report

**DOI:** 10.1097/MD.0000000000031420

**Published:** 2022-10-28

**Authors:** Jeong Eun Lee, Myeong Jin Kim

**Affiliations:** a Department of Anesthesiology and Pain Medicine, School of Medicine, Kyungpook National University, Kyungpook National University Hospital, Daegu, Republic of Korea.

**Keywords:** carbon dioxide, esophagectomy, pneumomediastinum

## Abstract

Surgery is the treatment of choice for esophageal cancer. Since the 1990s, minimally invasive esophagectomy (MIE) has been developed using videoscope. Although MIE lowers mortality by reducing postoperative complications, the risk of carbon dioxide (CO_2_) insufflation related complications still exists.

**Patient concerns:**

A 56-years-old male patient underwent elective MIE. The patient (body mass index, 15 kg/m^2^) had well-controlled hypertension, cardiomegaly, and severe emphysematous lungs. He had iatrogenic pneumothorax during central venous catheterization 3 weeks prior; however, the pneumothorax was resolved before surgery.

**Diagnosis:**

During thoracoscopic surgery, respiratory acidosis was not corrected despite rapid respiratory rate and positive end-expiratory pressure. Intrathoracic CO_2_ pressure was lowered from 12 to 8 mm Hg, and laparoscopic surgery was performed through the diaphragm in the reverse Trendelenburg position. In 15 minutes at this position, pulseless electrical activity with respiratory failure and high peak inspiratory pressure developed.

**Interventions:**

CO_2_ insufflation was stopped and drained as soon as hypotension developed. The patient was placed in the supine neutral position, and cardiopulmonary circulation was restored without further treatment.

**Outcomes:**

After the pneumomediastinum event, surgery was successfully performed. Respiratory acidosis due to CO_2_ insufflation was not corrected during surgery and the patient was transferred to intensive care unit without extubation. After 14 days, the patient was discharged without cardiopulmonary complications. However, the patient expired 2 years later due to cardiovascular disease.

**Lessons:**

In MIE, there is always a risk of catastrophic tension pneumomediastinum along with intravascular volume depletion, surgical position, and ventilatory strategy depending on the surgical characteristics.

## 1. Introduction

Esophageal cancer has the 6^th^ highest mortality rate worldwide, and surgical treatment is the primary treatment of choice. Open esophagectomy-related complications, such as tissue trauma, anastomosis leakage, cardiopulmonary stress response, and pneumonia contribute to the increase in mortality; therefore, the 5-years survival rate of esophageal cancer is still as low as 18%.^[1–4]^ Since the 1990s, videoscope-guided surgical techniques have enabled minimally invasive esophagectomies (MIE), which has minimized postoperative pain due to small incisions and helped experts improving surgical dissection and anastomosis techniques. As contrasted with open esophagectomy, MIE reduced the severe adverse impact on the patient’s daily life until 2 years. These results show that MIE can reduce the patients recovery time to return to daily life and postoperative progression of the chronic pain.^[5,6]^

To support successful MIE, intra-cavital gases such as carbon dioxide (CO_2_) are injected concomitantly to secure a surgical field for MIE. Depending on the MIE technique used, CO_2_ must be insufflated into both the abdominal and thoracic cavities during long-term surgical procedures. MIE can be challenging in maintaining hemodynamic variables without CO_2_ related complications. Insufflated gas-related complications vary from mild subcutaneous emphysema to severe gas embolism, pneumothorax, and pneumomediastinum (PM).^[7]^

We encountered a case of sudden cardiac collapse due to tension PM during a laparoscopic abdominal procedure after thoracoscopic esophageal dissection in the middle of MIE. Because the patient had severe emphysematous lung, the differential diagnosis was difficult to rule out tension pneumothorax, which has a different plan of treatment with PM. Therefore, we report a fatal course of tension PM and substantial risk during MIE.

## 2. Case presentation

**Figure 1.**
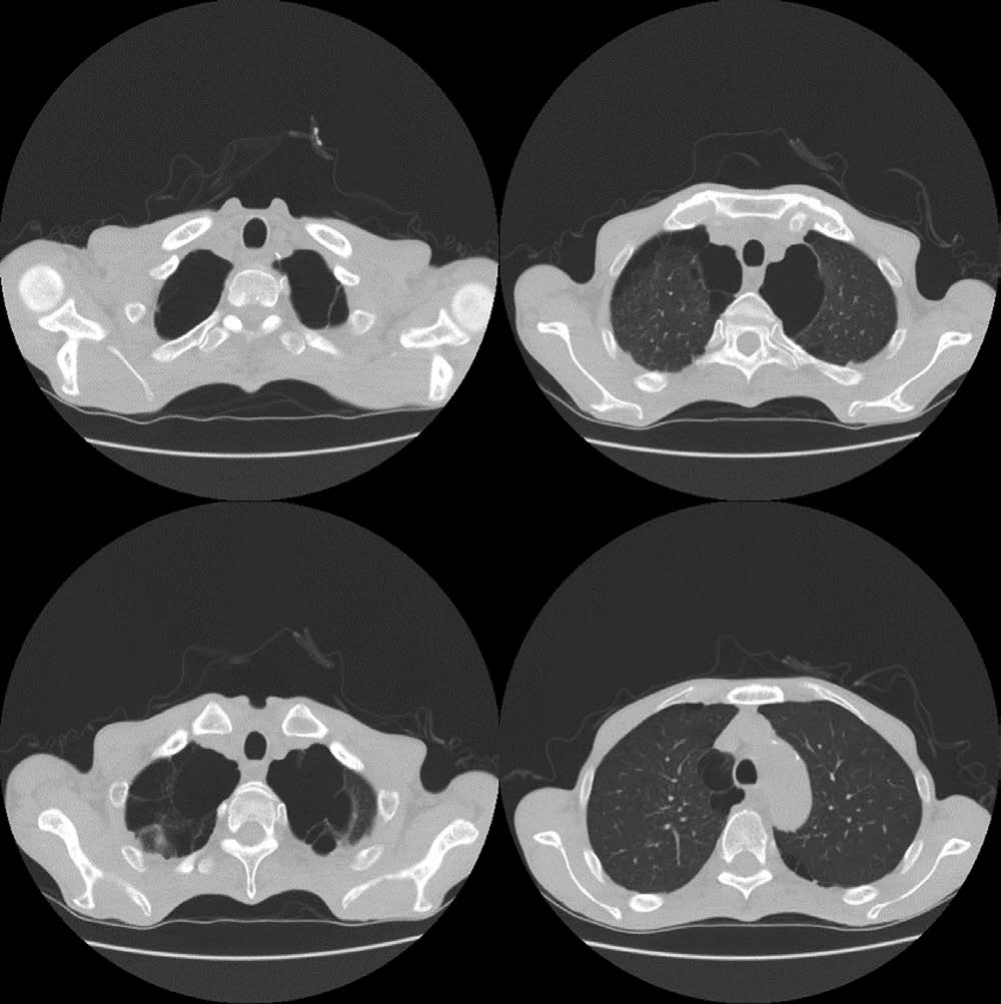
Preoperative chest computed tomography shows severe emphysematous lungs.

A 56-years-old male patient underwent elective esophageal cancer surgery with MIE. His height was 166.6 cm and weight was 41.6 kg (body mass index, 15 kg/m^2^). Five years ago, he was diagnosed with hypertension but had not taken any antihypertensive drugs due to low blood pressure 3 months before the diagnosis of esophageal cancer. Preoperative electrocardiogram showed a right bundle branch block and left anterior fascicular block. Chest radiography showed cardiomegaly (cardiothoracic [C/T] ratio, 68%); however, cardiac function was intact with mild mitral and tricuspid valve regurgitation on transthoracic echocardiography. The patient had emphysematous lungs and several huge bullae (Fig. 1). Iatrogenic pneumothorax developed during central venous catheter insertion 3 weeks before surgery. Therefore, a chest tube was inserted, for a week, to resolve the pneumothorax. There was no air leakage after removal of the chest tube. All laboratory test results were within normal ranges.

The neural integrity monitored endotracheal tube was inserted for intraoperative neural monitoring, and mechanical ventilation was set as follows: fraction of inspired oxygen 0.5, tidal volume 350 to 400 mL, respiratory rate 13 to 15 per minute, positive end-expiratory pressure 5 cmH_2_O. General anesthesia was maintained with desflurane and remifentanil. The surgery was initiated in the prone position, and extensive mediastinal dissection of the esophagus was performed using a right thoracoscopic approach with 2-lung ventilation. Standard thoracoscopic trocars were inserted and CO_2_ was insufflated under a maximal pressure of 12 mm Hg. After the specimen removal via thoracoscopy, the patient was placed in the supine position, and cervical lymphadenectomy was performed. After cervical lymphadenectomy, the reverse Trendelenburg position was raised to approximately 45° for the gastric stump laparoscopic abdominal resection. The patient already had uncontrolled high arterial CO_2_ pressure (64.6 mm Hg) due to the prone position and thoracoscopic surgery, and aggressive ventilation could not be applied because of severe emphysematous lungs. The anesthesiologist requested the surgeon to reduce the intraabdominal CO_2_ insufflation pressure due to respiratory acidosis (pH, 7.209), and the insufflation pressure was maintained at 8 mm Hg at all times in the reverse Trendelenburg position. Fifteen minutes after the gastric stump surgery, the blood pressure steeply decreased to 72/53, and the heart rate increased to 90 per minute. Pulse oximetry did not work simultaneously. Peak inspiratory pressure increased, end-tidal CO_2_ decreased (below 25 cm H_2_O), and the end-tidal CO_2_ curve was distorted. First, we suspected a tension pneumothorax caused by a previous iatrogenic event or large bullae. We stopped the procedure and performed auscultation; however, we could not approach the lungs appropriately because of the surgical field. During management, systolic blood pressure decreased at 50 s, the arterial blood pressure showed pulseless electrical activity, and gas insufflation was stopped immediately. Simultaneously, the surgeon drained all the gas through the laparoscopic ports. We used 100% oxygen (O_2_) and reduced the tidal volume and respiratory rate. As soon as all the insufflated gas in the abdomen was removed, the blood pressure gradually increased, and ventilation started to be as high as the setting tidal volume. Arterial blood gas analysis was performed after the restoration of the patient’s hemodynamic status, and the results were as follows: pH, 7.205; pCO_2_, 70.4 mm Hg; pO_2_ 71.1 mm Hg; and O_2_ saturation, 90.0%. After this event, as both lung sounds were clear, we proceeded with the operation. Further surgery was performed in the supine neutral position, and esophageal anastomosis in the neck was performed without any further complications. The results of arterial blood gas analysis and hemodynamic variables during surgery are summarized in Table 1.

**Table 1:** Patient monitoring variables and arterial blood gas analysis during surgery.

		Prone	Prone & Capnothorax	Prone to supine	Reverse Trendelenburg & capnoperitoneum	Tension pneumomediastinum	Neutral position & CO_2_ off	End of surgery
Patient monitoring	ETCO_2_ (mm Hg)	31	39	48	38	25	45	
	CVP (cmH_2_O)	6	11	11	9	15	9	
	CI	3.5	3.8	3.9	3.6	2.4	4.8	
	SVV	9	6	5	8	29	16	
Arterial blood gas analysis	pH	7.455	7.341	7.209	7.294	7.205	7.211	7.333
	pCO_2_ (mm Hg)	36.9	38.6	64.6	51.9	70.4	60.6	41.6
	pO_2_ (mm Hg)	270.3	91.7	103.5	268.9	71.1	195.3	191.9
	HCO_3_ (mmol/L)	25.9	20.4	21.5	22.6	22.6	20.6	21.1
	Base Excess (ECF) (mmol/L)	1.5	–5.4	–2.7	–1.9	–0.8	–4.1	–4.3
	Sodium (mmol/L)	134.3	135.4	135.1	133.8	134.7	135.5	134.6
	Potassium (mmol/L)	3.86	3.50	4.31	4.18	4.12	3.94	3.68
	Chloride (mmol/L)	104	111	107	105	104	106	105

CI = cardiac index, CVP = central venous pressure, ETCO_2_ = end tidal carbon dioxide, SVV = stroke volume variance.

**Figure 2.**
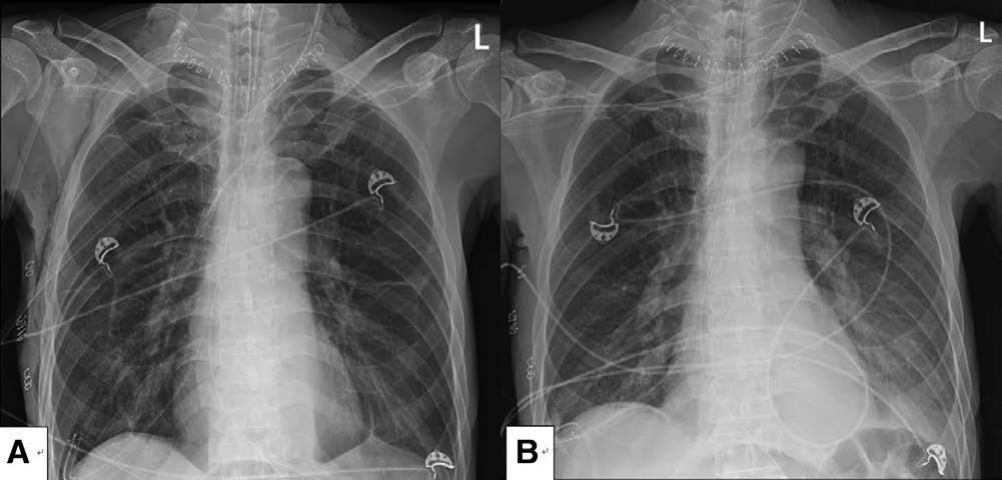
Postoperative chest radiography. A. Capnomediastinum on postoperative day 0. B. Extubation was performed on postoperative day 2.

The patient was transferred to the intensive care unit without extubation because of respiratory acidosis and postoperative pain control. Chest radiography performed after admission to the intensive care unit, showed slightly visible PM; however, chest computed tomography was not performed (Fig. 2). Extubation was performed on postoperative day 2 (Fig. 2). The patient did not show any abnormalities; therefore, he was discharged on postoperative day 14. However, he died of cardiovascular disease 2 years later. The family provided informed consent for publication and we anonymized the patient data.

## 3. Discussion

MIE using thoracoscopy or laparoscopy is generally considered a routine method. In this study, right thoracoscopic esophageal dissection was performed, and a gastric conduit was prepared using a laparoscope. After cervical lymph node dissection, anastomosis through the diaphragm was performed between the stomach and remnant esophagus using upper abdominal laparoscopy.^[8]^ In MIE, although CO_2_ insufflation is necessary to secure the field of view of the scope, unstable hemodynamic events can occur due to an increase in intrathoracic or intraperitoneal pressure during anesthetic management. Hemodynamic variables related to CO_2_ insufflation can be controlled if proper cardiovascular management is performed, such as maintaining minimal insufflation pressure, proper intravascular volume, and active vasoconstrictor use. However, there is always a risk of CO_2_-related pneumothorax or PM, as with other laparoscopic surgeries.

The insufflated CO_2_ easily tracks along a low-resistance dissected site into the defective mediastinum, including the sites of esophageal dissection or trans-hiatal anastomosis, and can develop tension PM even at low gas insufflation pressure. Besides CO_2_ insufflation pressure, other risk factors for tension PM are caused by the effect of restricted blood volume status, decreased compliance of the lung, reverse Trendelenburg surgical position, and ventilation strategy.^[9]^ Intraoperative fluid overload affects the incidence of acute lung injury after esophagectomy.^[10]^ Restrictive fluid management significantly reduces cardiac preload with intracavital CO_2_ insufflation, and decreased cardiac output leads to pulseless electrical activity in the case of tension PM. The intraoperative reverse Trendelenburg position also affects the hemodynamic status and reduces cardiac output by up to 50%.^[11–15]^ Additionally, positive end-expiratory pressure is frequently applied to improve ventilation in the presence of increased thoracic pressure owing to intra-abdominal gas insufflation. These factors in the emphysematous lung concomitantly cause a significant decrease in the cardiac output.^[16]^

Nevertheless, fatal events related to the tension type of PM are very rare because gas insufflation-related complications occur depending on the absorption rate of the gas used and the amount of gas that is removed.^[17]^ The occurrence of PM is mostly recognized by extraperitoneal gas insufflation, which is accompanied by subcutaneous emphysema and pneumothorax, resulting in electrocardiogram changes and an increase in end tidal CO_2_, airway pressure, and Pa CO_2_. If tension PM develops, treatment of PM can be adjusted by its source of air insufflation to the mediastinum, using treatment options such as mediastinoscopy, pericardiocentesis, or closed thoracostomy tubes in the case of pneumothorax. Considering MIE surgical procedure, the possibility of isolated tension PM always exists; however, this is the 1^st^ case report of cardiac arrest. If tension PM on MIE is not recognized in advance, its diagnosis is inevitably delayed.

MIE is a complicated surgery; therefore, it can be difficult to determine the cause of cardiovascular collapse occurring in hypovolemic patients during videoscopy. In this case, the blood pressure increased gradually as soon as CO_2_ insufflation was stopped. The gas was released after pulseless electrical activity, and tension PM was suspected. A distinct cardiac border was found on a chest radiograph taken immediately after surgery, which confirmed cardiac collapse due to isolated tension PM. Although rare, PM may develop without hemodynamic changes during laparoscopic surgery, and insufflated gas-related PM is reported to occur in approximately 2% of adult patients.^[18]^ Especially in the case of laparoscopic cholecystectomy, scant capnomediastinum was observed in approximately 40% of postoperative chest radiographs in the post-anesthetic care unit.

In MIE, PM should be differentially diagnosed, including pneumothorax, CO_2_ embolism, and subcutaneous emphysema. Pneumothorax can occur during alveolar rupture due to excessive straining during mechanical ventilation with a thoracoscope. The patient had emphysematous lungs and relatively low lung compliance. The case was difficult to diagnose because of multiple large bullae. Giant bullae can be mistaken for pneumothorax. As in our case, auscultation alone could not be used for the primary differential diagnosis of pneumothorax, chest radiography and lung USG are needed for differential diagnosis. Computed tomography scan can confirm the diagnosis, but it is difficult to perform due to cost, time, and circumstances that make it impossible to perform during surgery. Chest tube insertion is the treatment of choice for tension pneumothorax; however, in rare cases of misdiagnosis, misidentified giant bullae cause serious complications such as hemothorax due to rupture of the bullae through thoracotomy.^[19]^ In addition to pneumothorax, CO_2_ embolism develops relatively commonly during videoscope-guided surgery immediately when the Veress needle unintentionally enters the vein or parenchymal organ. Meanwhile, delayed CO_2_ embolism can occur at any time if the Veress needle penetrates an open blood vessel during surgery. However, a sudden decrease in capnography was not observed to suspect CO_2_ embolism in this case, the peak pressure increased to 40 cmH_2_O, and ventilation was not performed at the same time. Therefore, tension pneumothorax due to ruptured bullae or iatrogenic pneumothorax 3 weeks prior was considered the next greatest possibility. When tachycardia, high peak inspiratory pressure, insufficient tidal volume, desaturation, central venous pressure rises, and hypotension eventually leads to hypotension, typical symptoms of tension pneumothorax also appear. However, subcutaneous emphysema (crepitus), which often accompanies this condition, could not be confirmed because it was a surgical field. Fortunately, we confirmed PM with CO_2_ deflation and postoperative radiological examinations.

With recent advances in surgical and anesthetic techniques for MIE, surgical trials are also increasing for high-risk patients.^[20]^ There are many different surgical methods for MIE, and data on the incidence of PM in MIE are scarce. Sudden cardiac collapse due to tension PM during surgery is uncommon and it is difficult to make a differential diagnosis if it is not predicted. In particular, if there are multiple giant bullae, pneumothorax must also be identified, making the diagnosis more difficult. Based on this report, our case reports result suggests that the potential risk of tension PM should be fully recognized in MIE.

## Author contributions

**Conceptualization:** Jeong Eun Lee.

**Data curation:** Jeong Eun Lee, Myeong Jin Kim

**Formal analysis:** Jeong Eun Lee.

**Investigation:** Jeong Eun Lee.

**Methodology:** Jeong Eun Lee.

**Project administration:** Jeong Eun Lee, Myeong Jin Kim

**Validation:** Jeong Eun Lee.

**Visualization:** Jeong Eun Lee.

**Writing – original draft:** Jeong Eun Lee.

**Writing – review & editing:** Jeong Eun Lee.

## References

[1] Ferlay J Soerjomataram I Dikshit R. Cancer incidence and mortality worldwide: sources, methods and major patterns in GLOBOCAN 2012. Int J Cancer. 2015;136:E359–86. 25220842 10.1002/ijc.29210

[2] Dunst CM Swanström LL. Minimally invasive esophagectomy. J Gastrointest Surg. 2010;14(Suppl 1):S108–14. 19789930 10.1007/s11605-009-1029-x

[3] Biere SS van Berge Henegouwen MI Maas KW. Minimally invasive versus open oesophagectomy for patients with oesophageal cancer: a multicentre, open-label, randomised controlled trial. Lancet. 2012;379:1887–92. 22552194 10.1016/S0140-6736(12)60516-9

[4] Straatman J van der Wielen N Cuesta MA. Minimally invasive versus open esophageal resection: three-year follow-up of the previously reported randomized controlled trial: the TIME trial. Ann Surg. 2017;266:232–6. 28187044 10.1097/SLA.0000000000002171

[5] Malhotra GK Yanala U Ravipati A. Global trends in esophageal cancer. J Surg Oncol. 2017;115:564–79. 28320055 10.1002/jso.24592

[6] Mariette C Markar S Dabakuyo-Yonli TS. Health-related quality of life following hybrid minimally invasive versus open esophagectomy for patients with esophageal cancer, analysis of a multicenter, open-label, randomized phase III controlled trial: the MIRO trial. Ann Surg. 2020;271:1023–9. 31404005 10.1097/SLA.0000000000003559

[7] Herrerías JM Ariza A Garrido M. An unusual complication of laparoscopy: pneumopericardium. Endoscopy. 1980;12:254–5. 6448743 10.1055/s-2007-1021755

[8] Brierley RC Gaunt D Metcalfe C. Laparoscopically assisted versus open oesophagectomy for patients with oesophageal cancer-the randomised oesophagectomy: minimally invasive or open (ROMIO) study: protocol for a randomised controlled trial (RCT). BMJ Open. 2019;9:e030907. 10.1136/bmjopen-2019-030907 PMC6887040 31748296

[9] Hirvonen EA Poikolainen EO Pääkkönen ME. The adverse hemodynamic effects of anesthesia, head-up tilt, and carbon dioxide pneumoperitoneum during laparoscopic cholecystectomy. Surg Endosc. 2000;14:272–7. 10741448 10.1007/s004640000038

[10] Rucklidge M Sanders D Martin A. Anaesthesia for minimally invasive Oesophagectomy. Cont Educ Anaesth Crit Care Pain. 2010;10:43–7.

[11] Josephs LG Este-McDonald JR Birkett DH. Diagnostic laparoscopy increases intracranial pressure. J Trauma. 1994;36:815–9. 8015003 10.1097/00005373-199406000-00011

[12] Korell M Schmaus F Strowitzki T. Pain intensity following laparoscopy. Surg Laparosc Endosc. 1996;6:375–9. 8890423

[13] Bäcklund M Kellokumpu I Scheinin T. Effect of temperature of insufflated CO_2_ during and after prolonged laparoscopic surgery. Surg Endosc. 1998;12:1126–30. 9716765 10.1007/s004649900798

[14] Koivusalo AM Lindgren L. Effects of carbon dioxide pneumoperitoneum for laparoscopic cholecystectomy. Acta Anaesthesiol Scand. 2000;44:834–41. 10939696 10.1034/j.1399-6576.2000.440709.x

[15] Neudecker J Sauerland S Neugebauer E. The European association for endoscopic surgery clinical practice guideline on the pneumoperitoneum for laparoscopic surgery. Surg Endosc. 2002;16:1121–43. 12015619 10.1007/s00464-001-9166-7

[16] Gutt CN Oniu T Mehrabi A. Circulatory and respiratory complications of carbon dioxide insufflation. Dig Surg. 2004;21:95–105. 15010588 10.1159/000077038

[17] Worrell JB Cleary DT. Massive subcutaneous emphysema and hypercarbia: complications of carbon dioxide absorption during extraperitoneal and intraperitoneal laparoscopic surgery – case studies. AANA J. 2002;70:456–61. 12526151

[18] Alshahrani W Almaary J. Pneumomediastinum and ECG changes during laparoscopic Nissen fundoplication in a child; case report. Int J Surg Case Rep. 2020;77:830–3. 33395906 10.1016/j.ijscr.2020.11.034 PMC8253843

[19] Sf O Nm NA. A case of missed Giant Bullae emphysema diagnosed as pneumothorax. Med Health. 2017;12:90–3.

[20] Didiev MK Edilsultanova KA Rakitin TA. Features of anesthesia in minimally invasive surgery. J Pharm Res Int. 2021;33:49–55.

